# Synthesis, Characterization, *In vitro* and *In vivo* Evaluation of Pullulan-co-MAA Hydrogels Decorated with 5-fluorouracil for Oral-Colonic Delivery

**DOI:** 10.2174/0115672018380981251025052921

**Published:** 2026-02-13

**Authors:** Iqra Islam, Kaleem Ullah, Shujaat Ali Khan, Haya Yasin, Sheraz Ali, Ghulam Murtaza

**Affiliations:** 1 Department of Pharmacy, COMSATS University Islamabad, Abbottabad Campus, Abbottabad, Pakistan;; 2 Faculty of Pharmacy, Hamdard University, Islamabad Campus, Islamabad, Pakistan;; 3 Department of Pharmaceutical Sciences, College of Pharmacy and Health Sciences, Ajman University, Ajman, United Arab Emirates;; 4 Department of Pharmacy, COMSATS University Islamabad, Lahore Campus, Lahore, Pakistan

**Keywords:** Pullulan, pH-responsive hydrogels, characterization, in-vitro evaluation, MTT-assay, oral tolerability

## Abstract

**Introduction:**

Conventional treatment of colorectal cancer causes severe side effects, leading to non-compliance. Hydrogels are polymer-based oral drug delivery carriers that offer numerous advantages.

**Methods:**

This study describes the application of the free-radical polymerization approach for the synthesis of colon-targeted, pH-responsive, pullulan-co-MAA hydrogels to deliver 5-fluorouracil. The prepared hydrogels were characterized *in vitro*and *in vivo*.

**Results:**

*In vitro* characterizations confirmed successful crosslinking, a decrease in the drug’s crystallinity, a rough morphology, and the stable nature of the drug. Gel content, swelling index, porosity, and drug entrapment efficiency were increased with the increase in the concentration of polymer and monomer. The drug release followed a swelling study pattern; the cumulative drug release was increased with the increase in concentration of polymer (80.4% to 95.6%) and monomer (72.1% to 78.3%), while cross-linker negatively affected the drug release (62.2% to 53.5%) at pH 7.4. The results of the MTT-assay showed that blank hydrogels were cyto-compatible as all the cells displayed more than 95% viability, while drug-loaded hydrogels exhibited dose dose-dependent cytotoxic effect. Oral tolerability results showed that the hydrogel suspension was well-tolerated up to 4000mg/kg of body weight without altering hematology and serum chemistry profile or tissue histology of various organs.

**Discussion:**

The results confirmed successful hydrogel formation with effective 5-fluorouracil entrapment, enhanced thermal stability, and controlled drug release influenced by the concentrations of polymer, monomer, and crosslinker.

**Conclusion:**

According to findings, the fabricated hydrogels have promising potential to deliver 5-fluorouracil and other hydrophilic moieties into the colon.

## 1 INTRODUCTION

Colorectal cancer (CRC) is ranked as the third most prevalent cancer around the globe, with the second-highest mortality rate among all diseases [1, 2]. Conventional chemotherapy has shown significant limitations and severe systemic adverse effects against most cancers. These include compromised solubility, limited availability, and accumulation of the drug at the tumor site due to poor permeability, non-specific distribution, and damage to normal tissues [3-5]. The diverse physiological environment and pH gradient throughout the gastrointestinal tract (GIT) [6, 7], notably the stomach 1-3, highly acidic), small intestine (pH 5-7.5), large intestine (pH 6.8-7.8), and rectum 7.8-8 (basic pH) further makes it challenging for the drug to reach the target colon site [6, 7]. On the other hand, drugs for CRC treatment, when administered, must enter the systemic circulation which then leads to non-specific distribution and thus limits their pharmaceutical utilization [8, 9]. Furthermore, non-specificity of the anti-cancer drugs, sub-therapeutic concentration, and difficulty in reaching the desired target site are among the serious concerns that are faced by the formulation scientists in the conventional therapy approach [10, 11].

Keeping in view the current problems associated with the drug target site and conventional therapy approach, a colon-specific formulation can be used as an alternate option that has numerous advantages. This approach has the least invasiveness, pain sensation, ease of use and administration, cost effectiveness, and delivery of higher drug concentrations locally at the disease site to enhance efficacy and reduce the side effects [12, 13]. On the other hand, an ideal oral-colon-specific drug delivery carrier should overcome the challenges, such as preventing the non-target site drug release in the stomach and small intestine, and a smart triggering mechanism in response to the physiological changes in the colon [14]. So far, various DDS have been explored for oral-colon-specific drug delivery [15], among which hydrogels have served as a cornerstone due to interesting features such as biocompatibility, non-toxicity, biodegradability, flexible tunable nature, high swelling, resemblance with soft tissues in the swollen state, and sustained and controlled targeted drug release [16]. Technically, hydrogels are 3-dimensional polymeric networks that comprise crosslinking between polymers and monomers [17]. Among various hydrogels, pH-sensitive hydrogels are of prime importance. These hydrogels shrink or swell in response to the surrounding pH of various parts of the gastrointestinal tract (GIT) and thus release the drug at the desired site in a precise, sustained, or controlled pattern [18].

pH-responsive hydrogels fabricated utilizing natural or synthetic polymers with basic or acidic functional groups [19, 20]. Natural polymers are preferred due to their immense biocompatibility, biodegradability, and non-toxicity [21, 22]. In particular, polysaccharides such as pullulan have been extensively used in the synthesis of controlled and sustained release drug delivery systems [23].

Pullulan is a natural polymer obtained from the fungus *Aureobasidium pullulans*. It is a non-ionic, water-soluble homo-polysaccharide and possesses α-D-1, 6 linked maltotriosyl units in its structure [24]. Pullulan is recognized for its interesting features, such as biocompatibility, biodegradability, renewable origin, and highly hydrophilic nature. These features make pullulan a promising polymer for its wide use in various fields such as tissue engineering, cosmetics, food packaging, gene targeting, and most importantly, drug delivery [19, 20].

Methacrylic acid (MAA) is a monocarboxylic acid that is derived from acrylic acid [21]. It is biocompatible and easy to co-polymerize with different polymers and monomers. It is widely used in the preparation of pH-responsive hydrogels. The pH-responsive nature is attributed to its carboxylic acid, which ionizes to produce carbonyl ions. These ions possess a strong repulsive property that leads to significant swelling of the hydrogel network [22, 23].

5-Fluorouracil is useful for colonic cancer and is administered through an intravenous route. Additionally, oral administration is also limited by its rapid metabolism, GI toxicity, shorter half-life, and rapid clearance out of the body. To cope with the mentioned problems related to the drug and route of administration, there is a need to formulate a site-specific controlled or sustained drug delivery system for 5-fluorouracil [24].

This study uses a free-radical polymerization approach for the synthesis of colon-targeted, pH-responsive, pullulan-co-MAA hydrogels to deliver 5-fluorouracil. Ammonium persulfate (APS) and methylene-bis-acrylamide (MBA) were used as reaction initiators and cross-linkers, respectively.

## 2 MATERIALS AND METHODS

### 2.1 Materials

Pullulan was purchased from Spectrum Chemicals (USA). 5-Fluorouracil (~99% purity) was obtained from Thermofisher Scientific (Korea). All other chemicals, including ammonium persulfate, sodium hydroxide, hydrochloric acid, methacrylic acid, potassium hydrogen phosphate, and potassium chloride, were of analytical grade and acquired from DaeJung (Korea). N- N-methylene-bis-acrylamide was purchased from Sigma Aldrich (UK). Methanol was obtained from Merck (Germany).

### 2.2 Fabrication of pH-responsive PU-co-MAA Hydrogels

Previously reported free radical polymerization was used to prepare PU-co-MAA hydrogels with little modification [25]. Various concentrations of polymer, monomer, and cross-linker were used as mentioned in Table **1**. Briefly, pre-weighed quantities of polymer (pullulan), initiator (APS), and cross-linking agent (MBA) were dissolved in a predetermined volume of distilled water and stirred using a hot plate magnetic stirrer at 1000 rpm for 15 min to prepare a clear solution. Thereafter, Solution-B (initiator) was added gradually into MAA and stirred for 45 min with continued stirring at 1000 rpm, followed by the addition of Solution-A (pullulan). The mixture was allowed to stir again at 1000 rpm for 30 min to form a homogenous solution. In the final step, solution-C (MBA) was added drop-wise to the above-mentioned solution mixture with continuous stirring at 1000 rpm for 30 min, and the final volume was adjusted to 100 mL with distilled water. The whole process of solution preparation and mixing was performed at room temperature.

The finally obtained clear solution was covered and incubated, followed by a temperature rise to 50°C for 4 hrs., and lastly 55°C overnight. Clear gels with glossy textures and elastic properties were obtained after 24 hrs. The freshly prepared gels were carefully cut into 8 mm discs using specific molds and then washed by immersing in a 50% v/v water-ethanol solution for 1 hr. The washed gels were subjected to drying at room temperature until constant weight was attained. After complete drying, the gels were stored in pre-labeled air-tight jars.

### 2.3 Drug Loading

The swelling and diffusion method was employed for drug entrapment as reported previously [25]. The pH-responsive behavior of gels were considered, and a 5-fluorouracil solution was prepared in buffer pH 7.4. Pre-weighed dried hydrogel discs were immersed in 100 mL of the drug solution at 37°C for 48 hrs. Every six hours, the disc position was changed to confirm the even exposure of the hydrogel disc to the drug solution. Thus, the swollen hydrogel discs were carefully taken out, wiped, and dried. The process was conducted in triplicate.

### 2.4 Post-synthesis Characterization

#### 2.4.1 FTIR Spectroscopy

Fourier transform infrared (FTIR) analysis explored the interaction of the drug with ingredients. For this purpose, a 15 mg finely ground sample of polymer, monomer (liquid), drug, unloaded and drug-loaded hydrogels was used. The sample was scanned in the range of 500-4000 cm^-1^ using an FTIR spectrophotometer (Thermofischer-Scientific). A spectral matching approach was adopted to interpret the results [26].

#### 2.4.2 XRD Analysis

The X-ray diffraction (XRD) analysis tested crystalline features of pullulan and 5-fluorouracil after crosslinking. This analysis used a PAN analytical diffractometer by scanning the respective samples in the range of 10-100^◦^ at a scan rate of 2^◦^/min. The instrument was equipped with a copper (Cu) radiation source (1.542) and a voltage source of 40 KV [25].

#### 2.4.3 SEM Testing

Scanning electron microscopy (SEM) examined the external and cross-sectional surfaces of hydrogels by using a Tescan Vega-3 Scanning Electron microscope. The respective samples were mounted on an aluminum stub using double adhesive tape. The stubs were coated with gold to a thickness of ~300 Å using a high-vacuum evaporator and a gold sputter module for better conductivity. Scanning photomicrographs of both surface and cross-sectional parts were captured at various magnification powers [17].

#### 2.4.4 Thermal Studies

The effect of heat on the hydrogel’s stability was assessed through TGA and DSC studies using Q-500 series (WestSuccex-UK). The instrument was operated in standard mode, and the sample was analyzed in the range of 20-500˚C at a rate of 10˚C/min increase and 50mL/min nitrogen purging. The obtained data were analyzed using Universal Analysis 2000 (version 4.5A).

### 2.5 Post Synthesis In vitro Evaluation of PU-co-MAA Hydrogels

#### 2.5.1 Physical Appearance of Prepared Hydrogels

The final solution of reactants was closely observed visually to determine the effect of feed ratios on the physical appearance of the solution. The same observation was continued for the gels to determine the effect of feed ratios on texture, elasticity, and stickiness.

#### 2.5.2 Sol-gel Analysis

Sol-gel analysis was performed to determine the quantities of reactants consumed and crosslinking efficiency of the free radical polymerization technique used for the preparation of pullulan-co-MAA hydrogels. The soluble, unreacted contents formed after the polymerization reaction are known as “sol contents,” while the insoluble and reacted contents are known as “gel contents”. The dried discs were initially weighed and immersed in boiling distilled water for 4 hrs. in the Soxhlet apparatus. The unreacted reactants were eliminated throughout this time. After the aforementioned time of extraction, the gels were withdrawn from the apparatus and allowed to completely dry at room temperature until constant weight was attained by the gels. After completion of the drying process, the gels were weighed again [17]. The analysis was performed in triplicate

The following equations were used to determine sol fraction and gel fraction separately:

Gel fraction (%) = W_i_ – W_ext_ /W_i_ ×100

Where W_i_ and W_ext_ represent the weight of the hydrogel disc before and after extraction, respectively.

Sol fraction (%) = 100−gel fraction

#### 2.5.3 Porosity Measurements

The analysis was performed through a solvent replacement approach as reported earlier. For this purpose, 3 discs from each formulation were selected, and their diameter, thickness, and weight (Ra) were carefully checked. The weighed (Ra) discs were soaked in 100% ethanol for 48 hrs. and then removed. The surface solvent was blotted using filter paper and then weighed (Rb) precisely [27].

The percentage porosity was determined by applying the following equation:

Porosity (%) = R_b_−R_a_/ ρV × 100

Where ρ and V represent the density of absolute alcohol and the post-swelling volume of hydrogel, respectively.

#### 2.5.4 In vitro Degradation

The in-vitro degradation pattern of pullulan-co-MAA hydrogels was determined through the weight loss technique by exposing the gels to PBS pH 7.4, as reported earlier [28]. Pre-weighed, completely dried hydrogel discs were immersed in 10 mL of PBS pH 7.4 at 37^◦^C and 40 rpm in a shaking incubator. At pre-determined time intervals, the hydrogel discs were taken out, lyophilized at -80^◦^C, and weighed again. The experiment was performed in triplicate. The formula used to get the percentage weight loss is as follows:

Weight Loss (%) = W_1_ – W_2_/ W_1_ × 100

W_1_ and W_2_ are the sample weights before and after degradation, respectively.

#### 2.5.5 Swelling Study

The swelling experiment was performed at intestinal pH conditions. Pre-weighed, completely dried hydrogel discs were immersed in pre-labeled jars containing 100 mL of the relevant pH solution at 37°C. The hydrogel discs were removed from the jars at pre-determined time intervals, blotted with filter paper, and returned to the respective jars after weighing carefully. The experiment was continued until a constant weight was attained by the hydrogels. pH of the respective solution was maintained daily, while the analysis was performed in triplicate [25].

The swelling index was determined using the following equation:

SR (%) = W_b_ –W_a_/ W_a_ × 100

Where W_a_ indicates the weight of dried hydrogel discs and W_b_ indicates the weight of hydrogel discs after swelling in the respective media.

#### 2.5.6 Estimation of Drug Entrapment Efficiency (DEE)

For determining the amount of drug entrapped in the hydrogel discs, extraction by the solvent replacement procedure was used. For this purpose, completely dried drug-loaded hydrogel discs were immersed in pre-labeled jars containing 25 mL PBS (pH 7.4). The buffer solution was replaced every 24 hrs. with an equal volume of fresh buffer. The replaced buffer was analyzed through a UV-spectrophotometer at a 256 nm wavelength [18]. The procedure was carried out in triplicate.

#### 2.5.7 In-vitro Drug Release

The pH-dependent drug release study was carried out at pH 1.2, 6.8, and 7.4 to mimic stomach, intestine, and colonic conditions, respectively. The study was performed using the USP dissolution apparatus-II (Microtek Digital). The stirring speed was set at 100 ± 5 rpm, the temperature at 37 ± 0.5 ^◦^C, and 900 mL of the respective dissolution medium was filled in each basket. The apparatus was allowed to attain the desired temperature; thereafter, drug drug-loaded hydrogel disc was immersed in each basket. At pre-determined time intervals, a 5 mL sample was taken from each basket and analyzed at 256 nm using a UV-spectrophotometer. The withdrawn sample was replaced with an equal volume to maintain the sink conditions [25]. The study was performed in triplicate.

Drug Concentration was calculated through the following formula:

Y = MC + B

Where: Y is the solution's absorbency, M is the identified concentration's standard curve's angle, C is the estimated concentration, and B is the curve's cut-off.

#### 2.5.8 Kinetic Modeling of In vitro Drug Release

To determine the 5-fluorouracil release order and mechanism, the in-vitro drug release data were analyzed through various kinetic models, including Zero order, First order, Higuchi, and Korsmeyer-Peppas [29].

Zero-order

Qt = K0t

First order

Log Qt = log Q0 − K1t

Higuchi model

Qt = KHt 1/2

Korsmeyer Peppas model

M_t_/M_0_ = K_3_t^n^

#### 2.5.9 Cell Viability Assay

The cell viability (MTT) assay was performed with little modifications from the previous approach [24] to determine the cytocompatibility of formulations against healthy (FHC) and malignant (HCT-116) cell lines. Briefly, Minimum Essential Medium (MEM) was used to seed cell suspension (1×10 ^5^ cell concentration) in a 24-well plate. The well plates were incubated at 37°C for 24 hrs with 95% relative humidity and 5.0% CO_2_ concentration. Following MTT dye staining, the viable cells were again treated for 2 hrs. The percentage of viable cells was determined by measuring the mixture at 570 nm using a microplate reader after adding dimethylsulfoxide (DMSO) to remove the MTT dye. "Non-treated cells" functioned as the "negative control," "free drug" served as the "positive control," and "5-fluorouracil loaded hydrogels" served as the “treatment group” [30, 31].

### 2.6 In-vivo Studies

#### 2.6.1 Oral Tolerability Study and Safety Evaluation of Pullulan-co-MAA Hydrogels

The study was meticulously conducted following the guidelines specified by the Laboratory Animal Science Association, 2009 [32].

##### 2.6.1.1 Ethical Approval

The study design was evaluated by the Research Ethics Committee (REC), COMSATS University Islamabad (Abbottabad campus), Pakistan, to ensure safety, ethical, and hygiene protocols. The proposed study design was approved by the committee and allotted an ethical approval number: Phm.Eth/FA17-CSM10/18-010-74. This study complies with internationally accepted standards for animal research, following the 3Rs principle. The ARRIVE guidelines were followed for reporting experiments involving live animals and promoting ethical research practices.

##### 2.6.1.2 Procurement and Acclimatization of Animals

A total of 16 rabbits (8 male and 8 female) with an average age of 2-3 years and body weight of 2-2.2 kg were selected for the study protocol. The rabbits were acquired from the university’s animal house. The rabbits were kept in clean and dry wooden cages with free access to food and water. All the animals were closely monitored for their water and food intake, urination, salivation, reflex action, signs and symptoms of illness, and death for 7 days.

##### 2.6.1.3 Final Selection of Animals, Maximum Tolerance Dose (MTD) Calculation, and Administration

In general, the MTD method is used to evaluate the oral tolerability study of hydrogels because no lethal dose (LD_50_) can be calculated. For this purpose (MTD calculation), 04 rabbits (02 male and 02 female) with an average 2 - 22.2 kg body weight were chosen. Formulation PU-3 (containing the highest quantity of polymer) was finely crushed, and its suspension was prepared in 15 mL of distilled water and administered to rabbits in the range of 200-4200 mg/kg in 02 divided doses using a feeding tube connected to a 50cc disposable syringe. The concentration of hydrogel powder in suspension was increased at a frequency of 150 mg while keeping the volume of distilled water constant. A total of 12 rabbits (06 male and 06 female) divided into 2 groups (03 male and 03 female) with 2-2.2 kg average body weight were selected for administration of MTD to determine oral tolerability. Group I was assigned the tag of control, while Group II was assigned the tag of treatment. Each rabbit of both groups was individually marked on the ear for easy identification. All the rabbits were kept fasted for 15 hrs before administration of 1^st^ dose with free access to water. The control group was administered normal saline while the treatment group was administered 4200 mg/kg of body-weight hydrogel suspension in two divided doses. The rabbits were closely monitored for water and food intake, trembling, salivation, urination, diarrhea, and excretion, signs and symptoms of illness and death. The dosing was continued for 7 days. On day 8^th^, blood samples were collected from the marginal and jugular veins of all rabbits. The samples were preserved in pre-labeled ethylenediaminetetraacetic acid (EDTA) tubes, while a small portion was left clotted for hematology and serum chemistry profiling. Thereafter, all the animals were sacrificed hygienically, and various organs (heart, kidney, liver, stomach, and colon) were carefully separated without causing any damage. Each organ was separately preserved in pre-labeled jars containing 100 mL buffered formalin. The organ and blood samples were delivered to Ali Labs, Islamabad, Pakistan, where hematology, serum chemistry, and histopathology analysis were performed under the supervision of a qualified pathologist [17, 26].

### 2.7 Statistical Analysis

The data of all the experiments performed in triplicate are presented as mean ± standard deviation (SD). T-test was applied on sol-gel, swelling, drug loading, drug release, *in vitro* degradation, and MTT-assay data. For comparison of multiple groups, ANOVA was used. P-value ≤ 0.05 was considered statistically significant. All the graphs were drawn using OriginLab (version 9.0), while images were produced through Adobe Photoshop (version 2015.0.0).

## 3 RESULTS AND DISCUSSION

### 3.1 Formation and Physical Appearance of Hydrogels

Pullulan-MAA solution was visually observed to note the changes occurring during the synthesis, as exhibited in Fig. (**1A**-**D**). The final solution of reactants appeared clear and translucent initially, but turned milky color with a slight turbidity later. No bubble formation was observed during the initial 24 hours of hydrogel formation; however, some of them turned into a yellow hue due to different polymeric concentrations. Formulations CR-CR3 (with the highest concentrations of cross-linker) looked to be compact and rigid due to the high crosslinking ratio of the newly formed hydrogels.

### 3.2 Post-synthesis Characterization

#### 3.2.1 FTIR

FTIR was performed for the drug, polymer, monomer, unloaded hydrogels, and drug-loaded hydrogels, as shown in Fig. (**2**). IR spectra of 5-fluorouracil (Fig. **2a**) were characterized as: a peak at 3119 cm^−1^ is assigned to the NH stretching. The observed bands of (C=O) were found at 1719 cm^−1^ and 1645 cm^–1,^ respectively. Peak at 1243 cm^–1^ was attributed to the C-F stretching band, while the band at 1348 cm^−1^ was indicative of pyrimidine compound vibrations, whereas bands at 1243 cm^−1^ and 1180 cm^−1^ were assigned to the C–O and C–N vibrations, respectively. Peaks at 1245 and 809 cm^−1^ were attributed to C-H in-plane deformation and C-H out-of-plane deformation, respectively [33]. In the case of polymer (Fig. **2b**), the broad peak was found in the region of 3226 cm^−1^ due to stretching of the O–H functional group. The peak at 2935 cm^−1^ showed the presence of a C–H bond. Another peak was observed at 1008 cm^−1^ , which is assigned to the glycosidic (C–O–C) bond stretching [34].

The peaks in FTIR spectra of MAA (Fig. **2c**) in the range of 1600-1780 cm^− 1^ were attributed to the stretching of C=O and C=C, respectively, which confirms the presence of the functional group COOH. The bands at 1250 cm^− 1^ and 1140 cm^− 1^ are indicative of CO and the carbonyl bond C–O–C, respectively [35]. In unloaded Pullulan-co-MAA hydrogels (Fig. **2d**), a peak was found at 2933 cm^−1^ , which showed the presence of -CH stretching. A large peak at 1688 cm^−1^ is indicative of a COOH group, which may be present because of MAA. The peaks found at 1159 cm^−1^ and 1253 cm^−1^ were assigned to the C–O bond formation between MBA and Pullulan [34]. In the case of 5-fluorouracil-loaded Pullulan-co-MAA hydrogels (Fig. **2e**), peak shifts in the region of 3392 cm^−1^ as compared to pure 5-fluorouracil, which may be due to NH stretching vibration. The sharp peaks observed at 1683 cm^−1^ and 1167 cm^−1^ indicate the stretching of carbonyl and C-N vibrations. It was concluded that the drug was successfully entrapped and dispersed in the polymeric framework of PU-co-MAA hydrogels.

#### 3.2.2 XRD Analysis

The XRD diffractogram of 5-fluorouracil exhibited small peaks in the range of 12-24° and a major sharp peak at 29° followed by some minor peaks. The presence of these peaks justified the crystalline nature of 5-fluorouracil, as shown in Fig. (**3a**) [36]. In the case of pullulan Fig. (**3b**), a broad diffraction peak was observed in the range of 18-20°, demonstrating the amorphous nature [19]. On the other hand, Pullulan-co-MAA hydrogels Fig. (**3c**) exhibited a broad peak in the range of 19-21°, which justifies the successful crosslinking of the polymer as well as a further increase in amorphousness of the polymer [34].

The XRD patterns of 5-fluorouracil-loaded hydrogels (Fig. **3d**) exhibited different diffractograms in comparison with those of pure 5-fluorouracil, pullulan, and unloaded hydrogels. The characteristic sharp peaks of pure 5-fluorouracil (as observed in Fig. **3A**) were decreased significantly in the diffractogram of 5-fluorouracil-loaded hydrogel. The reason for this decrease in crystallinity may be attributed to the fact that in our study, 5-fluorouracil was loaded in the newly formed hydrogels using a swelling and diffusion approach. Post-entrapment, the reduction in peak intensity, broadening, or disappearance indicates decreased crystallinity. In this approach, the drug is first dissolved in a suitable solvent or buffer before loading. Upon dissolution in buffer, usually, the drug loses or decreases its crystallinity and leading to molecular dispersion in the hydrogel network, which is evident in the XRD pattern. Our obtained results are in fair agreement with the earlier study conducted by Reddy *et al*., while investigating a pectin-based dual-responsive hydrogel system for 5-fluorouracil [37].

#### 3.2.3 SEM

The SEM indicated the structural characteristics of hydrogels as shown in Fig. (**4A**-**D**). The photomicrographs observed at different magnification powers gave a different appearance. Unloaded hydrogel discs showed smooth, compact surfaces with cracks and numerous pores when observed at 20 μm. Close-up magnification (100x to 1000x) revealed a rough and grainy surface that offers an area for solute trapping, water retention, and drug entrapment. Capillaries and uneven porous morphology were seen in drug-loaded hydrogel discs, which have an impact on how quickly drugs are released from hydrogels. These pores are thought to be the areas of water permeation and the locations where external stimuli interact with them [38]. It concluded that drug-loaded PU-co-MAA hydrogels exhibited the best structural morphology with numerous pores, which further led to the enormous swelling and *in vitro* drug release.

#### 3.2.4 Thermogravimetric Analysis (TGA)

The TGA thermogram of 5-fluorouracil (Fig. **5a**) showed weight loss in two phases. The initial weight loss of approximately 8% was observed in the range of 50-215°C due to evaporation of moisture content, followed by another weight loss of approximately 47% in the range 220–330°C due to decomposition [33]. Pure pullulan (Fig. **5b**) displayed initial weight loss below 100°C which is attributed to evaporation of surface moisture, while the weight loss around 300°C is assigned to degradation. The polymer skeleton degradation was observed in the range of 250–370°C, similar to the thermal behavior of polysaccharides, where the onset of degradation was observed at 240°C and maximum decomposition around 290°C [19]. The thermogram of both unloaded and 5-fluorouracil-loaded hydrogel (Figs. **5c** and **5d**) was different and showed more stability in comparison with the thermograms of individual components. In the case of unloaded hydrogels, a slight peak was observed around 250°C, which is assigned to the degradation of the pullulan backbone, while 5-fluorouracil-loaded hydrogels displayed a peak around 290°C that is attributed to the decomposition of the drug.

#### 3.2.5 DSC

The DSC thermogram of pure drug displayed a sharp endotherm peak at approximately 285ºC that is assigned to the melting temperature, followed by decomposition around 317ºC (Fig. **6a**). This result is in agreement with the previously reported data [39]. In the case of pullulan (Fig. **6b**), two broad endothermic peaks were observed at 92.3°C and 283.7°C which are assigned to evaporation of the surface or contained moisture and melting decomposition, respectively [19]. The thermogram of unloaded hydrogel (Fig. **6c**) displayed a slight endothermic peak around 250°C that could be assigned to the melting of pullulan content, while in the case of drug-loaded hydrogels Fig. (**6d**), there is no distinct endothermic peak that indicates the higher thermal stability of the hydrogel network and molecular dispersion of the drug [33].

### 3.3 Post-synthesis In-vitro Evaluation of Pullulan-co-MAA Hydrogels

#### 3.3.1 Sol-gel Analysis

The analysis was performed to assess the solution and gel content of the prepared hydrogels with different ratios of pullulan, methacrylic acid, and methylene bisacrylamide as presented in Supplementary Fig. (**1**). The gel fraction of the formulations with various quantities of MAA (MA1, MA2, and MA3) was significantly increased (*P* = 0.002) with an increase in the concentration of MAA due to an increase in the crosslinking density of hydrogels. Asad *et al*. reported similar results, showing that an increase in MAA concentration led to an increase in gel fraction and improved crosslinking profile of hydrogels [17].

The increase in polymer concentration (PU1, PU2, and PU3) had a significant (*P* = 0.000) impact on the gel fraction of hydrogels. The increased polymer concentration enhances the number of active sites for free radical polymerization, leading to crosslinking and gel formation [40]. The formulations with an increased ratio of cross-linker (CR1, CR2, and CR3) led to a significant (*P* = 0.001) increase in gel fraction. Increased MBA concentration causes molecules to attach to one another more frequently, resulting in a more compact structure and leading to an escalation in the gel fraction rather than the sol fraction [25].

#### 3.3.2 Porosity Measurement

For all formulations, porosity measurements were carried out to assess how well a medium penetrated the drug carrier system. The porosity of the hydrogel affects swelling and the drug loading phenomenon. According to the results, it was observed that porosity was significantly increased with the increase in the concentration of pullulan (*P* = 0.001) and MAA (*P* = 0.001) content, as shown in Supplementary Fig. (**2**). The increased polymer and monomer content led to an increase in the total number of ionizable hydrophilic hydroxyl (–OH) and carboxylic acid (COOH) groups that, upon ionization, lead to repulsion in polymeric chains and hence increased porosity. On the other hand, a higher crosslinking agent concentration causes more monomer–polymer crosslinks, which significantly decrease porosity (*P* < 0.001). Suhail *et al*. reported similar results while preparing pH-sensitive hydrogels for colon-targeted drug delivery [41].

### 3.4 Drug Entrapment Efficiency (DEE)

The increased concentrations of pullulan, MAA, and MBA had a significant impact on drug loading in fabricated hydrogels, as shown in Table **2**. The drug entrapment was significantly increased with the increase in concentration of polymer (*P* = 0.003) and monomer (*P* < 0.000), and drug entrapment efficiency was significantly increased. Pullulan and MAA contain –OH and COOH functional groups, respectively, that upon ionization cause a higher charge density. Eventually, a higher swelling ratio and drug entrapment were observed. However, the effectiveness of drug loading and entrapment was negatively related to cross-linker concentration (*P* = 0.002) as a more dense and compact hydrogel network is formed with an enhanced ratio of MBA. Our results are by the previously reported studies [25, 42].

### 3.5 Swelling and Drug Release

#### 3.5.1 Impact of pH on Swelling and Drug Release

The newly prepared hydrogels exhibited significant pH-responsive behavior at acidic and basic pH conditions. It was observed that hydrogels displayed high swelling and drug release at basic pH conditions (pH 6.8 & 7.4) in comparison with the acidic pH (1.2).

#### 3.5.2 Influence of Pullulan Content on Swelling and 5-fluorouracil Release

The effect of hydrogel content (polymer, monomer, and cross-linker) on selling and drug release is shown in Figs. (**7A**-**D** and **8A**-**D**), respectively. Formulations with various concentrations of pullulan (P1-P3) were studied for swelling and drug release at pH 1.2, 6.8, and 7.4. It was observed that swelling was significantly increased with the increase in pullulan content at pH 1.2 (*P* < 0.000), 6.8 (*P* < 0.000), and 7.4 (*P* < 0.000). The 5-fluorouracil release also followed a similar trend, and drug release was increased at pH 1.2 (*P* = 0.004), 6.8 (*P* = 0.001), and 7.4 (*P* = 0.001), as shown in Table **2**.

The behavior of pullulan is attributed to the presence of hydrophilic –OH groups. The hydrophilic properties of pullulan might be primarily responsible for the hydrogel's capacity to absorb water. This is a result of the interaction between pullulan and water molecules. Free -OH present in pullulan, which leads to an increase in water retention property, becomes available as pullulan concentration is raised. Hydrogen bonds, if formed by the –OH group of pullulan, are attributed to water absorption from the surrounding environment, resulting in swelling of hydrogels [19].

#### 3.5.3 Impact of MAA Content on Swelling and 5-fluorouracil Release

Formulations with various contents of MAA (MA1-MA3) displayed different swelling indices at acidic and basic pH conditions. At pH 1.2, there was a significant decrease in swelling (*P* = 0.002) and % drug release (*P* = 0.002) with the increase in MAA content. In contrast, swelling and drug release were increased at pH 6.8 (*P* = 0.001) and 7.4 (*P* = 0.001) with the increase in MAA content (Table **2**). MAA possesses an ionizable –COOH group in its structure that is responsible for its pH-responsive behavior. At pH 1.2, the –COOH group remains deprotonated and hence the hydrogel network remains collapsed, resulting in low swelling and drug release. Above pKa of –COOH group (pH 4.6), protonation takes place, resulting in the formation of negatively charged carbonyl ions (COO^-^) that lead to ionic repulsion. This repulsion in turn causes the polymeric chains to expand, leading to increased swelling and drug release. A similar behavior of MAA was reported previously while investigating guar gum-MAA hydrogels for metformin HCl [25].

#### 3.5.4 Effect of MBA Content on Swelling and 5-fluorouracil Release

The swelling of formulations containing various contents of MBA (C1-C3) was significantly decreased at pH 1.2 (*P* = 0.002), 6.8 (*P* < 0.000), and 7.4 (*P* < 0.000). Similarly, drug release was also decreased significantly (Table **2**) at pH 1.2 (*P* = 0.001), 6.8 (*P* < 0.000), and 7.4 (*P* < 0.000). This decrease in swelling and drug release is attributed to the typical behavior of MBA. By increasing the concentration, the crosslinking density of the hydrogel is increased, leading to the formation of a more dense, stiff, and compact polymeric network with less porosity and mesh size. This, in turn, limits the interaction of the hydrogel network with the surrounding swelling or dissolution medium, resulting in decreased swelling index and % drug release at both acidic and basic pH conditions. Our findings follow the results reported previously while investigating Sericin-based hydrogels for controlled delivery of acyclovir, where MBA was used as a crosslinking agent [26]. Although mesh size calculations and polymer network modeling could further elucidate the swelling and drug release behavior, such analyses were beyond the scope of the current study. Future investigations incorporating these approaches may provide deeper mechanistic insights. In addition, Supplementary Fig. (**3**) represents the swelling index of formulations MA-3, PU-3, and CR-3.

### 3.6 Drug Release Kinetics

An important factor in the release of drugs is the chemical structure of a polymeric network, which has a significant impact on how it swells. Different kinetic models were used to determine drug release patterns from the data obtained from *in vitro* drug release, *i.e*. zero order, first order, Higuchi, and Korsmeyer-Peppas. The results are presented in Table **3**, which indicates that all formulations followed first-order kinetics since the value of the coefficient of regression R^2^ was found to be higher in first-order than the zero-order values, suggesting that all the formulations displayed sustained release behavior. The R^2^ values in the Higuchi model were found to be higher than 0.5, which suggests that the drug release was based on diffusion. The "n" number for Korsmeyer-Peppas was found to be smaller than 0.45 in the majority of formulations, indicating Fickian behavior. The drug release from the formulations in our study is found to be concentration-dependent [17]. The kinetic models of formulation PU-1-CR-3 are shown in Supplementary Fig. (**4**). Furthermore, Supplementary Fig. (**5**) presents the cumulative drug release at pH 1.2, 6.8, and 7.4.

### 3.7 In vitro Degradation Assay

The degradation behavior of pullulan-co-MAA hydrogels defined the characteristics of surface degradation, which mostly resulted from the breakage of the hydrolytic polymer. Significant weight loss was observed in formulations containing various contents of pullulan (*P* < 0.000), MAA (*P* = 0.001), and MBA (*P* = 0.004), as shown in Supplementary Fig. (**6**). It was found that hydrogels absorb significant solution due to their complex hydrophilic structure and undergo degradation through erosion, hydrolysis, and other degradation mechanisms. In addition, as the immersion time of the hydrogels increased, the rate of weight loss also increased. This is mainly attributed to the fact that, as the cross-linked junctions are broken, it permits the complete disintegration of the cross-linked polymeric chains, resulting in overall weight loss of the hydrogels [28].

### 3.8 MTT Assay

The cytocompatibility and cytotoxic potential of blank hydrogels, and free 5-fluorouracil and 5-fluorouracil-loaded hydrogels were investigated against FHC (healthy) and HCT-116 (cancerous) cell lines. As depicted in Fig. (**9**), blank hydrogels were proven to be cytocompatible and showed high cell viability (> 95%) against both cell lines. In the case of free 5-fluorouracil and 5-fluorouracil-loaded hydrogels, a dose-dependent cytotoxic potential was observed as the % cell viability was reduced with increasing doses per well. FHC cell lines showed 75.03% and 78.12% cell viability against free 5-fluorouracil as well as 5-fluorouracil-loaded hydrogels respectively, at the concentration of 20 μg/mL. In the case of HCT-116, 22.38% of cells were viable against free 5-fluorouracil, while 23.93% of cells were viable against 5-fluorouracil-loaded hydrogels at 20 ug /mL concentration. It has been found that 5-fluorouracil is much more toxic in free form as compared to encapsulated form in the hydrogel matrix. 5-fluorouracil-loaded hydrogels showed a dose-dependent cytotoxic effect in the range of 10 to 20 μg/mL when compared to the free drug. This may be because of the drug’s entrapment in the polymeric network of hydrogels, leading to the sustained release of the drug. The analysis proved the cytotoxic effect of 5-fluorouracil even after loading in Pullulan-co-MAA hydrogels. Our findings are in agreement with a previous study conducted that reported similar results [23, 43].

### 3.9 Oral Tolerability Study and Safety Profiling of Pullulan-co-MAA Hydrogels in Rabbits

#### 3.9.1 Calculation of Maximum Tolerance Dose (MTD)

The MTD is the highest dose of a medication that a test subject may tolerate without experiencing morbidity or death. In the present study, hydrogel suspension in the range of 200-4200 mg/kg of body weight was administered in divided doses to the rabbits. At a dose of 4200 mg/kg of body weight, symptoms of irregular breathing, tachycardia, excessive urine, and loss of appetite became apparent, probably as a result of the stomach becoming full. Neither morbidity nor mortality was observed while calculating MTD. After taking everything into account, the MTD was decided upon as 4000 mg/kg of body weight.

#### 3.9.2 Monitoring of General Conditions

During the whole course of the study, no morbidity or mortality was observed in the control or the experimental group. The general conditions of hair, eyes, eye slits, oral cavity, and teeth were observed to be normal. Breathing and reflex action to light and sound were also found to be normal. Food and water intake, salivation, and lacrimation were also found to be normal. The frequency and color of urine and feces were also normal and free of blood and pus. Lastly, it was determined that a dose of 4000mg/kg of body weight was well tolerated by all the animals.

#### 3.9.3 Hematology and Serum Chemistry

Hematology and serum chemical profiling were done on blood samples from the treatment and control groups. After oral delivery, there is a chance that the unreacted substances will leak out of the hydrogel network and produce compatibility problems.

The serum chemistry and complete blood count (CBC) profiling were conducted on day 7. The CBC analysis aimed to sort out any blood abnormality caused by the hydrogel suspension in comparison with the standard control group. Hematology and serum chemistry analyses were performed for the determination of renal and liver functions. Results of hematology and serum chemistry analysis for both the groups (test and control) are presented in Tables **4** and **5**, respectively. It is evident from the obtained results that kidney and liver function, as well as blood values of the treatment group, were fairly normal and in close range with the control group. Metabolite concentration for both groups was also found to be in a close range and hence proved to be fairly normal. These results demonstrate the biocompatibility of newly produced hydrogels and allow for further *in vivo* studies in animal models [44].

#### 3.9.4 Histopathological Examination

Histopathological micrographs of heart, stomach, liver, kidney, and colon of control and treatment groups are described in the Fig. (**10a**-**e**). Fig. (**10a**) illustrates the heart of the control and treatment group (pullulan-co-MAA hydrogels). The micrographs of the heart revealed healthy cardiac muscles and cardiac myocytes that lacked any evidence of enlargement or hypertrophy. Myocardium, endocardium, and pericardium were found to be normal. In case of stomach micrographs (**10b**), there was no ulceration or bleeding, and the mucosa was found to be normal. The micrographs of liver (**10c**), displayed no necrosis or degeneration. There were no signs of macrophages, neutrophils, lymphocytes infiltration, and hyperemia, as well as no hypertrophy of hepatic sinusoids. The hepatic lobules and hepatic cords were fairly in normal order. The size of the kidneys (**10d**) was also demonstrated to be normal in both groups. The nephrons were undoubtedly apparent, and there was no evidence of bleeding. The glomerulus had a definite space around it and was in normal shape. The colon (**10e**) was examined for histopathological abnormalities and confirmed to be normal. There were no signs of bleeding, mucosal damage, ulceration, or necrosis. The treatment and control groups did not differ histo-pathologically overall. The overall results are well supported by the studies conducted in the past, while investigating the oral tolerability of chitosan-based hydrogels for polyoxometalate and gelatin-based hydrogels for colonic delivery of oxaliplatin in rabbits, respectively [31, 44].

## CONCLUSION

Pullulan-based pH-responsive hydrogels with different feed ratios of polymer, monomer, and cross-linker were successfully formulated using the free radical polymerization technique. The successful crosslinking and 5-fluorouracil entrapment were confirmed through post-synthesis characterization techniques. FTIR confirmed the successful interaction of polymer and monomer, as well as 5-fluorouracil entrapment. XRD patterns proved the decrease in crystallinity and molecular dispersion of the drug in the hydrogel network. SEM micrographs revealed an uneven surface morphology, while cross-sectional micrographs depicted a glassy appearance with sharp edges. TGA and DSC results showed that the thermal stability of individual components was increased after crosslinking. The swelling, drug loading, and drug release were increased with the increase in concentration of polymer and monomer while the crosslinking agent displayed an inverse behavior. The prepared hydrogels proved to be more responsive at pH 6.8 and 7.4 in comparison with pH 1.2. The blank hydrogels were cytocompatible, while free 5-fluorouracil and 5-fluorouracil-loaded hydrogels exhibited dose-dependent cytotoxic potential against FHC and HCT-116. Oral tolerability results revealed that the hydrogel suspension was well-tolerated up to 4000 mg/kg of body weight without causing any morbidity or mortality. Based on the obtained *in vitro* and *invivo* results, pullulan-co-MAA hydrogels can be claimed as a potential pH-responsive carrier system for oral-colon delivery of 5-fluorouracil as well as other hydrophilic drugs.

## Figures and Tables

**Fig. (1):**
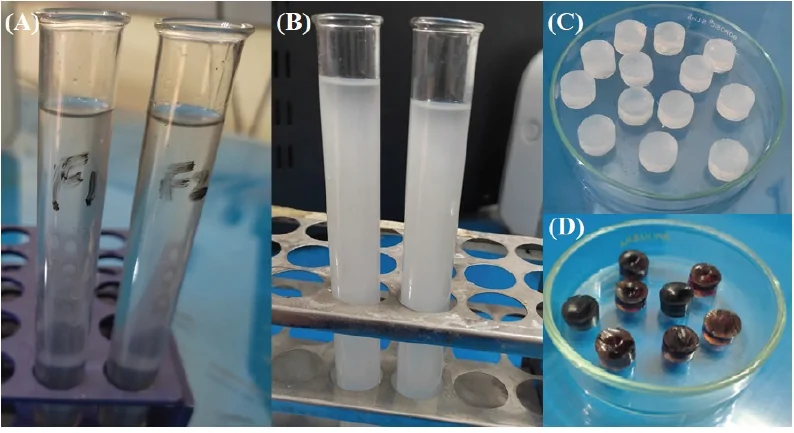
(**a**) Final solution of hydrogel components, (**b**) newly prepared hydrogels, (**c**) newly prepared hydrogels in disc form, (**d**) oven-dried hydrogel discs.

**Fig. (2):**
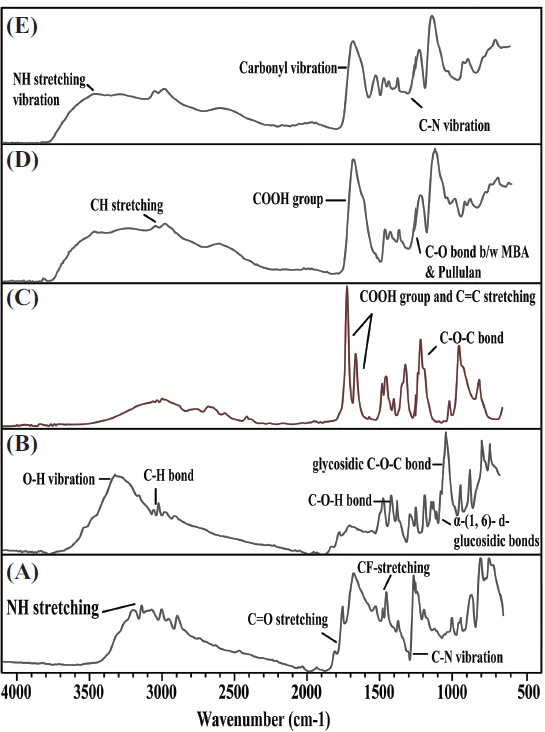
(**a**) Pure 5-fluorouracil, (**b**) pullulan, (**c**) MAA, (**d**) unloaded hydrogel, (**e**) 5-fluorouracil loaded hydrogel.

**Fig. (3):**
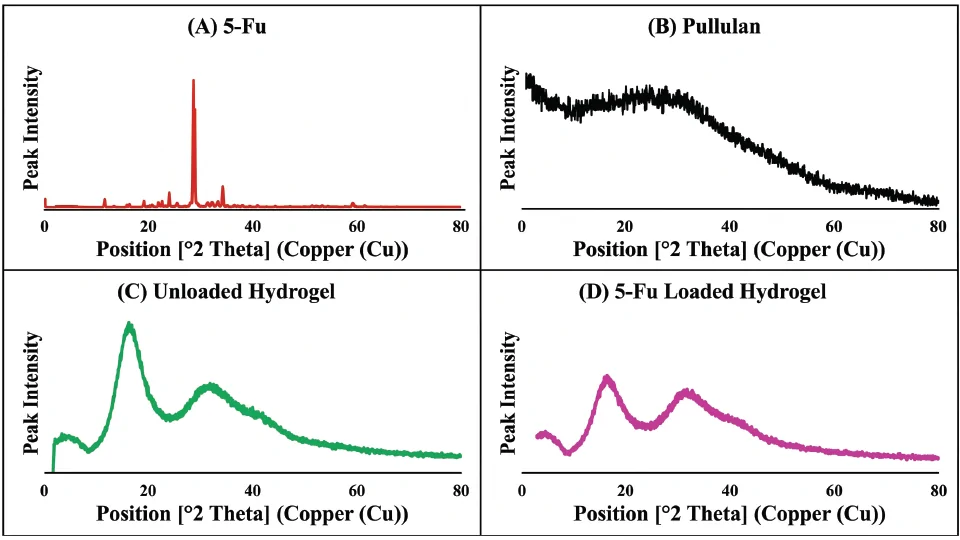
XRD patterns of: (**a**) 5-fluorouracil, (**b**) Pullulan, (**c**) unloaded hydrogel, (**d**) 5-fluorouracil loaded hydrogel.

**Fig. (4):**
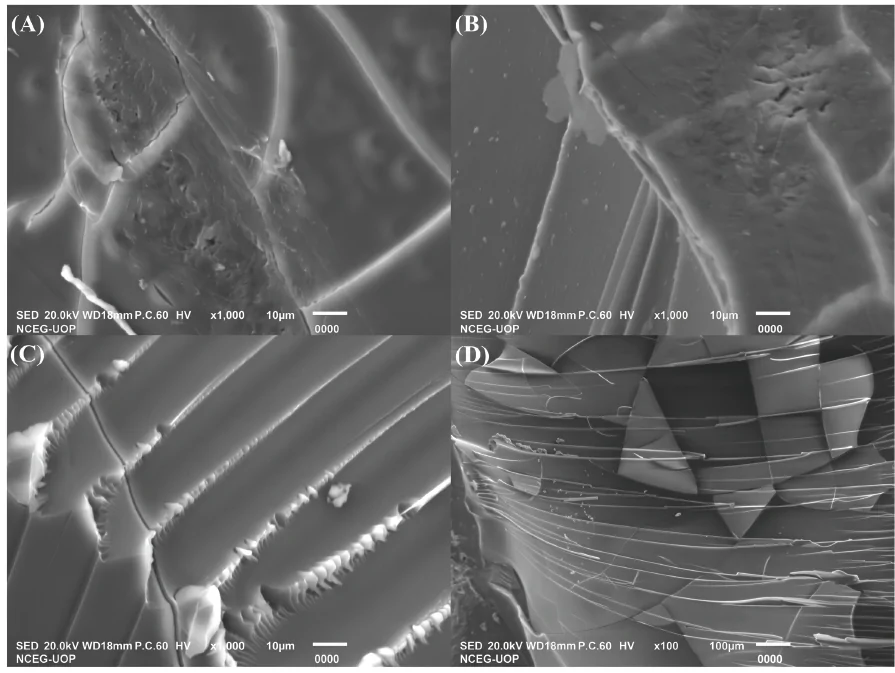
SEM pictures of external surface (**a** and **b**) and cross-sectional surface (**c** and **d**) of hydrogels.

**Fig. (5):**
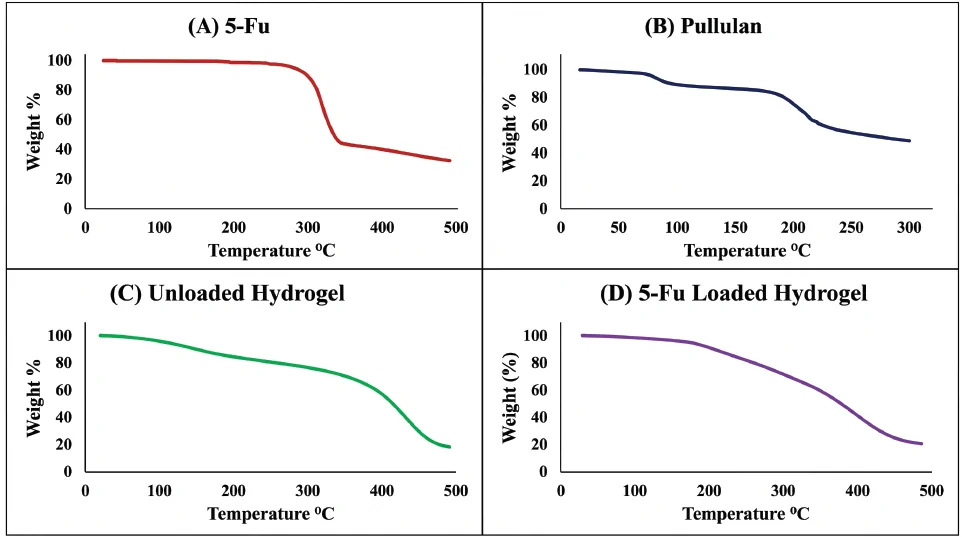
TGA graphs of: (**a**) 5-fluorouracil, (**b**) pullulan, (**c**) unloaded hydrogel, (**d**) 5-fluorouracil loaded hydrogel.

**Fig. (6):**
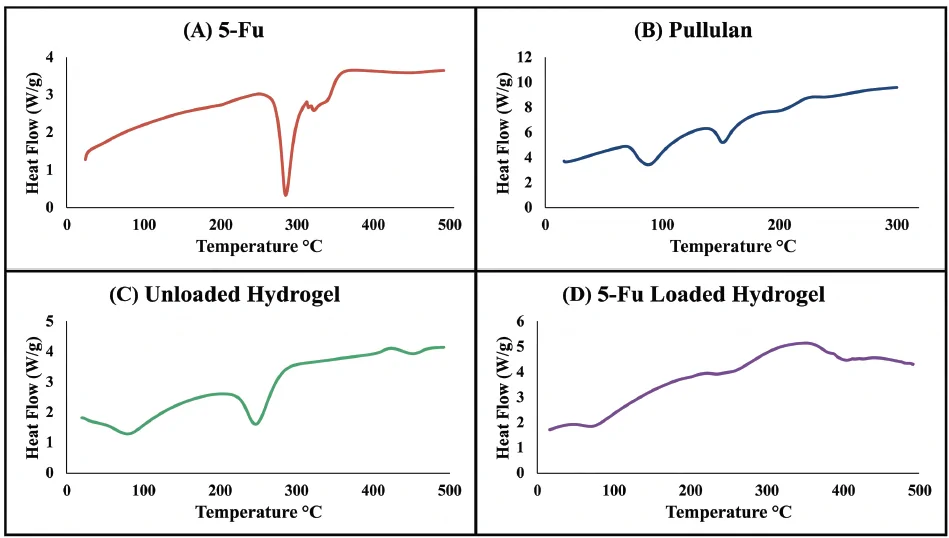
DSC graphs of: (**a**) 5-fluorouracil, (**b**) pullulan, (**c**) unloaded hydrogel, (**d**) 5-fluorouracil loaded hydrogel.

**Fig. (7):**
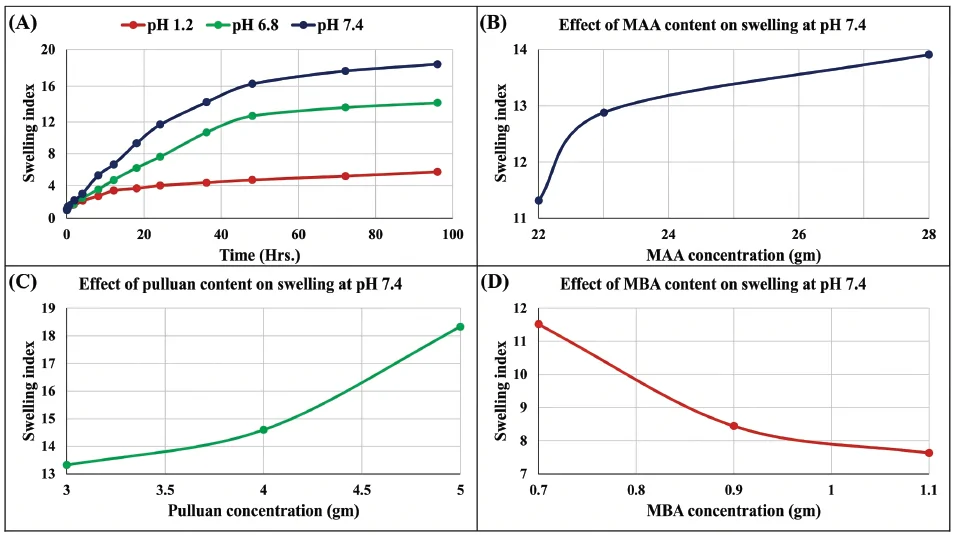
(**a**) swelling profile of formulation PU-3 at pH 1.2, 6.8 & 7.4 (**b, c, d**) effect of MAA, pullulan, and MBA content on swelling at pH 7.4.

**Fig. (8):**
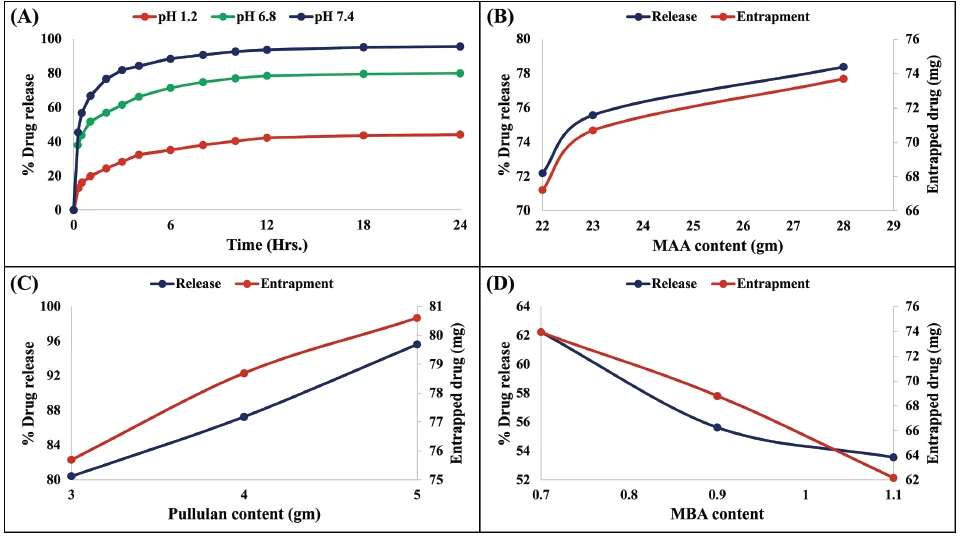
(**a**) drug release profile of formulation PU-3 at pH 1.2, 6.8 & 7.4 (**b, c, d**) effect of MAA, pullulan, and MBA content on 5-fluorouracil entrapment and release at pH 7.4.

**Fig. (9):**
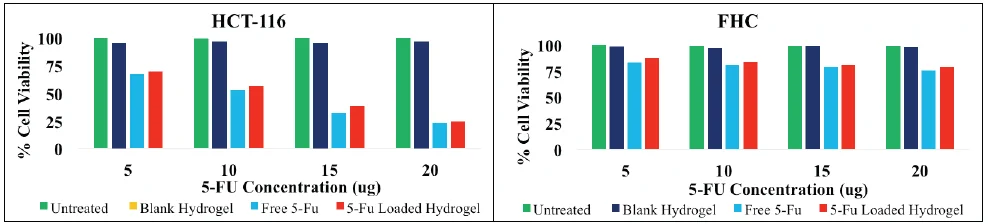
Graphical representation of % Cell viability against HCT-116 and FHC cell line.

**Fig. (10):**
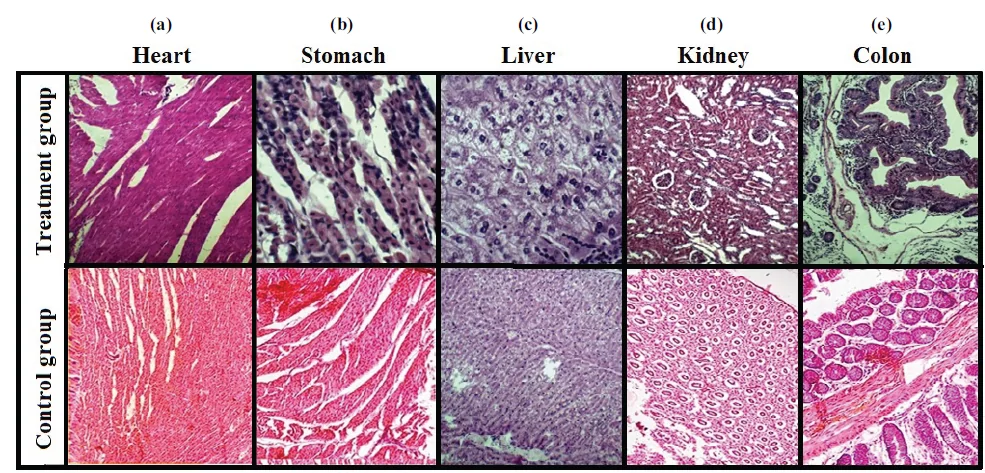
Histopathological micrographs of: (**a**) heart, (**b**) stomach, (**c**) liver, (**d**) kidney, (**e**) colon.

**Table 1:** Feed scheme of PU-MAA hydrogels.

**Formulation Code**	Pullulan (g/100g)	MAA (g/100g)	MBA (g/100g)	APS (g/100g)
MA1	2.0	22	0.5	1.0
MA2	2.0	23	0.5	1.0
MA3	2.0	28	0.5	1.0
PU1	3.0	28	0.5	1.0
PU2	4.0	28	0.5	1.0
PU3	5.0	28	0.5	1.0
CR1	2.0	28	0.7	1.0
CR2	2.0	28	0.9	1.0
CR3	2.0	28	1.1	1.0

**Table 2:** Amount of 5-fluorouracil entrapped (per disc) and % 5-fluorouracil released at pH 1.2, 6.8 & 7.4.

**Formulation Code**	**Entrapped 5-fluorouracil (mg/Hydrogel Disc) ± SEM**	**5-fluorouracil Released (%) ± SEM**		
		**pH 1.2**	**pH 6.8**	**pH 7.4**
MA-1	67.21 ± 0.92	22.81 ± 1.38	66.32 ± 2.71	72.11 ± 1.62
MA-2	70.36 ± 1.21	19.93 ± 2.19	69.15 ± 2.84	75.63 ± 1.75
MA-3	73.73 ± 2.37	16.91 ± 2.44	71.26 ± 2.16	78.37 ± 1.08
PU-1	75.58 ± 1.89	33.77 ± 2.81	71.82 ± 3.11	80.46 ± 2.35
PU-2	78.41 ± 2.15	38.46 ± 1.79	77.84 ± 2.49	87.32 ± 1.14
PU-3	80.61 ± 3.02	44.58 ± 1.47	80.22 ± 2.63	95.61 ± 2.96
CR-1	73.94 ± 2.77	15.62 ± 2.95	51.75 ± 1.27	62.28 ± 1.25
CR-2	68.82 ± 1.48	12.81 ± 2.10	48.47 ± 1.82	55.64 ± 1.77
CR-3	62.26 ± 1.73	10.63 ± 1.63	46.11 ± 1.03	53.52 ± 1.89

**Table 3:** Drug release kinetic models of prepared formulations.

**Sample Codes**	**pH**	**Zero-order**	**First Order**	**Higuchi Model**	**Korsmeyer-Peppas**
		**R^2^**	**R^2^**	**R^2^**	**N**
PU-1	1.2	0.82	0.85	0.96	0.29
	6.8	0.81	0.89	0.95	0.26
	7.4	0.69	0.79	0.88	0.20
PU-2	1.2	0.79	0.82	0.94	0.28
	6.8	0.68	0.79	0.87	0.20
	7.4	0.57	0.74	0.78	0.19
PU-3	1.2	0.72	0.77	0.90	0.28
	6.8	0.65	0.76	0.86	0.17
	7.4	0.54	0.81	0.76	0.15
MA-1	1.2	0.83	0.85	0.96	0.24
	6.8	0.85	0.92	0.96	0.41
	7.4	0.71	0.79	0.89	0.40
MA-2	1.2	0.85	0.87	0.97	0.26
	6.8	0.82	0.88	0.94	0.38
	7.4	0.74	0.84	0.91	0.33
MA-3	1.2	0.87	0.88	0.98	0.24
	6.8	0.77	0.86	0.93	0.30
	7.4	0.75	0.85	0.92	0.26
CR-1	1.2	0.83	0.84	0.96	0.28
	6.8	0.78	0.82	0.93	0.49
	7.4	0.70	0.77	0.89	0.44
CR-2	1.2	0.81	0.82	0.95	0.31
	6.8	0.77	0.82	0.92	0.53
	7.4	0.74	0.81	0.91	0.43
CR-3	1.2	0.76	0.77	0.93	0.40
	6.8	0.78	0.82	0.93	0.58
	7.4	0.70	0.77	0.89	0.47

**Table 4:** Hematological investigations of control and treatment groups.

**Parameter**	**Control (Group I)**		**Treatment (Group II)**	
	**Male (*n*=3)**	**Female (*n*=3)**	**Male (*n*=3)**	**Female (*n*=3)**
pH	7.19 ± 0.05	7.06 ± 0.05	7.28 ± 0.04	7.41 ± 0.06
Hemoglobin (g/L)	109.12 ± 0.58	113.38 ± 0.92	112.32 ± 1.73	111.76 ± 1.09
RBCs × 10^12^/L	6.23 ± 0.09	5.81 ± 0.15	5.28 ± 0.17	6.05 ± 0.03
MCV	64.65 ± 0.66	60.48 ± 0.53	67.16 ± 0.72	61.35 ± 0.19
MCH (Pg)	23.73 ± 0.02	22.5 ± 0.005	21.35 ± 1.23	24.76 ± 0.09
MCHC g/dL	30.76 ± 1.26	32.25 ± 0.09	33.49 ± 2.25	35.56 ± 1.08
Neutrophils × 10^9^/L	4.08 ± 0.05	4.17 ± 0.07	4.25 ± 0.03	4.28 ± 0.05
Lymphocytes ×10^9^/L	2.16 ± 0.07	2.23 ± 0.09	2.51 ± 0.04	2.39 ± 0.14
Eosinophils × 10^9^/L	8.16 ± 0.05	8.28 ± 0.08	7.98 ± 0.09	8.09 ± 0.05
Platelets × 10^9^/L	355.17 ± 3.32	342.56 ± 1.54	361.60 ± 1.78	351.32 ± 2.55
Basophils × 10^9^/L	0.29 ± 0.03	0.37 ± 0.04	0.35 ± 0.05	0.45 ± 0.02

**Table 5:** Serum analysis profile of control and treatment groups.

**Parameter**	**Control (Group I)**		**Treatment (Group II)**	
	**Male (*n*=3)**	**Female (*n*=3)**	**Male (*n*=3)**	**Female (*n*=3)**
Albumin (g/L)	50.13 ± 0.15	53.62 ± 0.95	53.79 ± 1.15	55.23 ± 0.69
ALP (U/L)	131.3 ± 0.08	133.28 ± 0.92	135.86 ± 1.25	132.55 ± 0.24
ALT (U/L)	78.10 ± 1.51	82.71 ± 0.09	80.56 ± 0.16	84.56 ± 0.53
AST (U/L)	60.56 ± 0.35	62.09 ± 0.66	61.72 ± 2.09	63.23 ± 1.78
Total Protein (g/L)	67.17 ± 0.52	72.21± 0.34	71.32 ± 0.11	70.71 ± 0.72
Urea (mmol/L)	15.82 ± 0.47	12.59 ± 1.76	14.56 ± 0.05	13.15 ± 0.83
Creatinine (µmol/L)	152.67 ± 1.78	154.16 ± 2.26	158.78 ± 0.09	156.22 ± 0.69
Uric Acid (mg/dL)	3.55 ± 0.25	3.64 ± 0.09	3.76 ± 0.11	3.61 ± 0.09
Glucose (mmol/L)	7.98 ± 0.67	8.06 ± 1.36	8.25 ± 0.05	8.68 ± 0.13
Globulin (g/L)	20.55 ± 0.35	21.19 ± 1.89	24.06 ± 0.55	20.91 ± 0.14
Cholesterol (mmol/L)	105.27 ± 2.14	109.16 ± 0.65	104.58 ± 1.76	106.18 ± 1.13

## Data Availability

The data and supportive information are available within the article.

## LIST OF ABBREVIATIONS

ALP: Alkaline Phosphatase
ALT: Alanine Aminotransferase
ANOVA: Analysis of Variance
APS: Ammonium Persulfate
AST: Aspartate Aminotransferase
CBC: Complete Blood Count
CRC: Colorectal Cancer
DDS: Drug Delivery System
DEE: Drug Entrapment Efficiency
DMSO: Dimethyl Sulfoxide
DSC: Differential Scanning Calorimetry
FTIR: Fourier Transform Infrared Spectroscopy
GIT: Gastrointestinal Tract
LD50: Lethal Dose 50%
MAA: Methacrylic Acid
MBA: N,N'-Methylenebisacrylamide
MCH: Mean Corpuscular Hemoglobin
MCHC: Mean Corpuscular Hemoglobin Concentration
MCV: Mean Corpuscular Volume
MTD: Maximum Tolerated Dose
MTT: 3-(4,5-dimethylthiazol-2-yl)-2,5-diphenyl tetrazolium bromide
PBS: Phosphate-Buffered Saline
PU: Pullulan
RBC: Red Blood Cells
SD: Standard Deviation
SEM: Scanning Electron Microscopy
TGA: Thermogravimetric Analysis
UV: Ultraviolet
XRD: X-ray Diffraction
