# Supplementary material for: Synthesis, Characterization, In vitro and In vivo Evaluation of Pullulan-co-MAA Hydrogels Decorated with 5-fluorouracil for Oral-Colonic Delivery
Source: Curr Drug Deliv. 2026 Feb 13;23(8):966–83. doi: 10.2174/0115672018380981251025052921 (PMC13684975; doi:10.2174/0115672018380981251025052921)
Supplement: Supplementary file 1 [file CDD-23-8-966_SD1.pdf]

SUPPLEMENTARY MATERIAL

Synthesis, Characterization, *In vitro* and *In vivo* Evaluation of Pullulan-co-MAA Hydrogels Decorated with 5-fluorouracil for Oral-Colonic Delivery

Iqra Islam[1], Kaleem Ullah[2], Shujaat Ali Khan[1,*], Haya Yasin[3,*], Sheraz Ali[1] and Ghulam Murtaza[4,*]

[1]*Department of Pharmacy, COMSATS University Islamabad, Abbottabad Campus, Abbottabad, Pakistan;* [2]*Faculty of Pharmacy, Hamdard University, Islamabad Campus, Islamabad, Pakistan;* [3]*Department of Pharmaceutical Sciences, College of Pharmacy and Health Sciences, Ajman University, Ajman, United Arab Emirates;* [4]*Department of Pharmacy, COMSATS University Islamabad, Lahore Campus, Lahore, Pakistan*

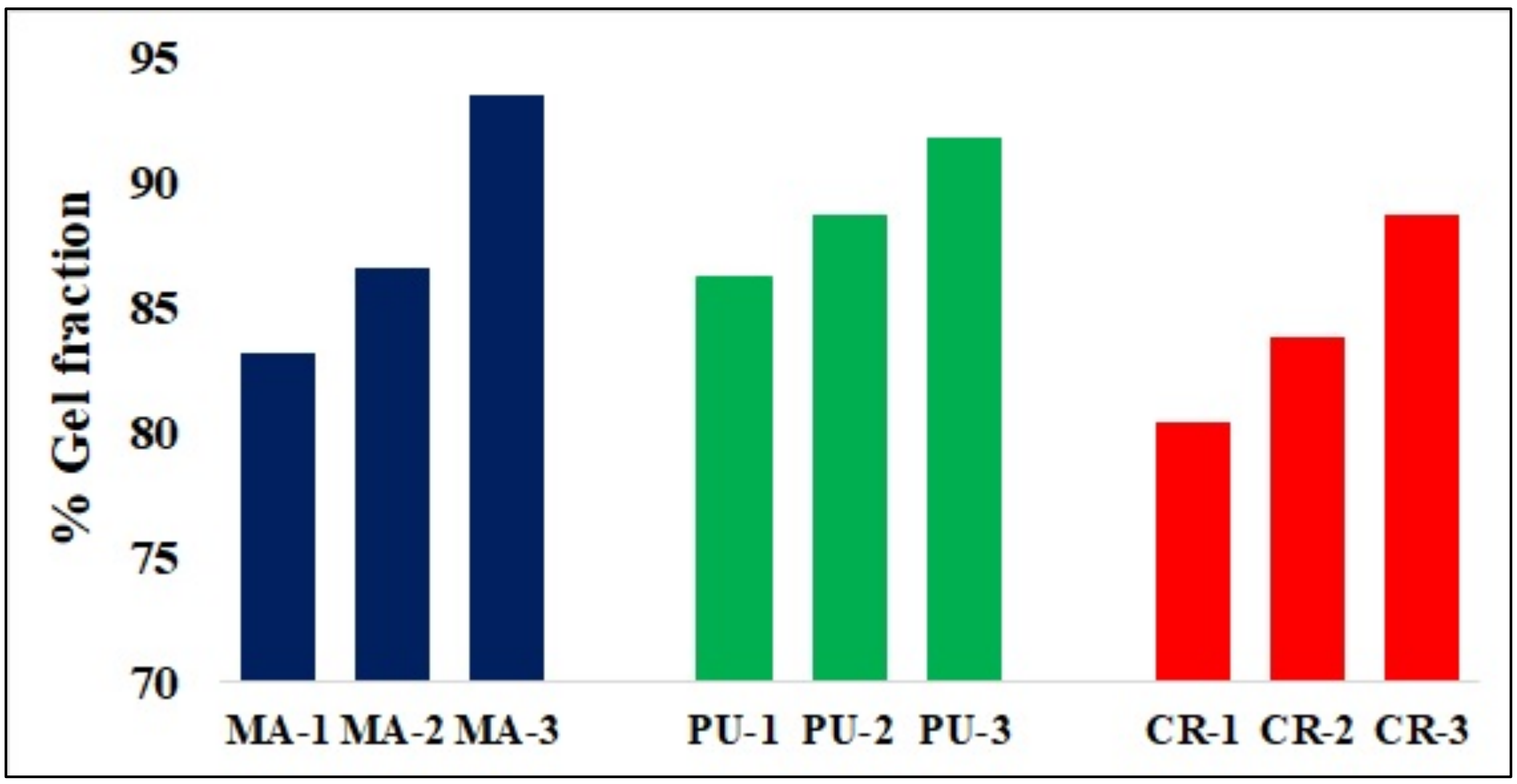

Fig. (S1). Effect of MAA, pullulan and MBA content on gel fraction of pullulan-co-MAA hydrogels.

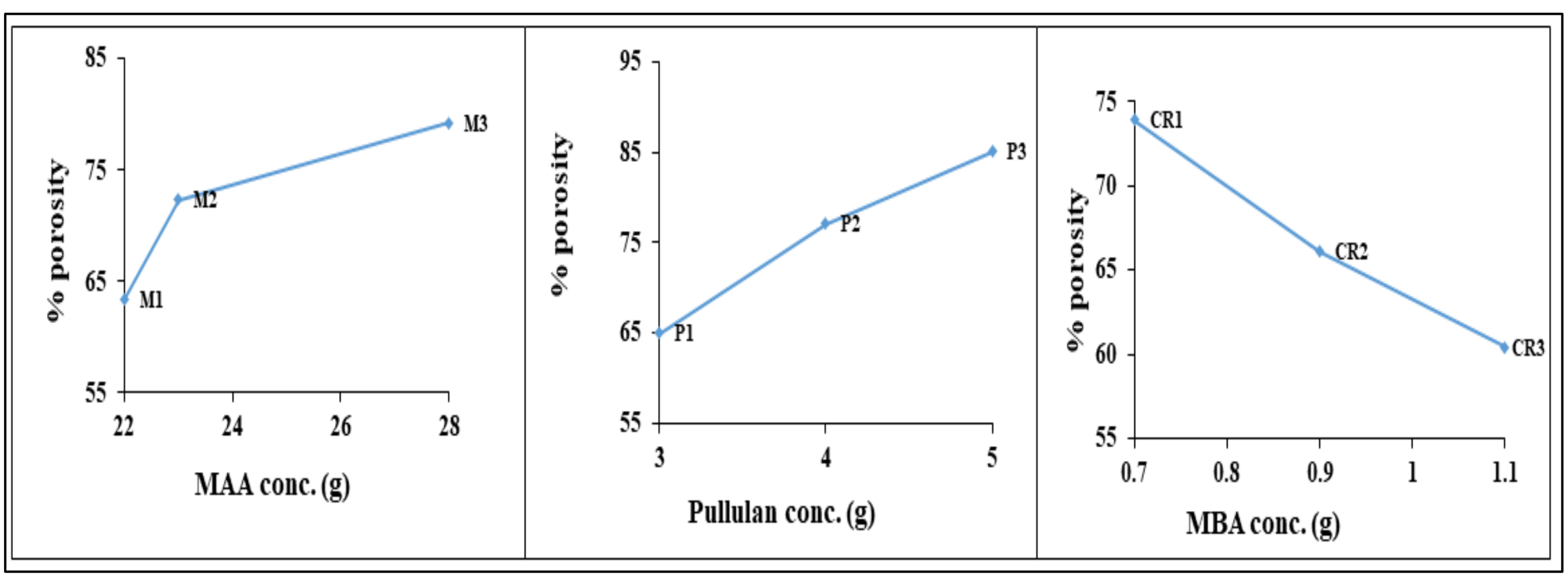

Fig. (S2). Effect of MAA, pullulan and MBA content on porosity of pullulan-co-MAA hydrogels.

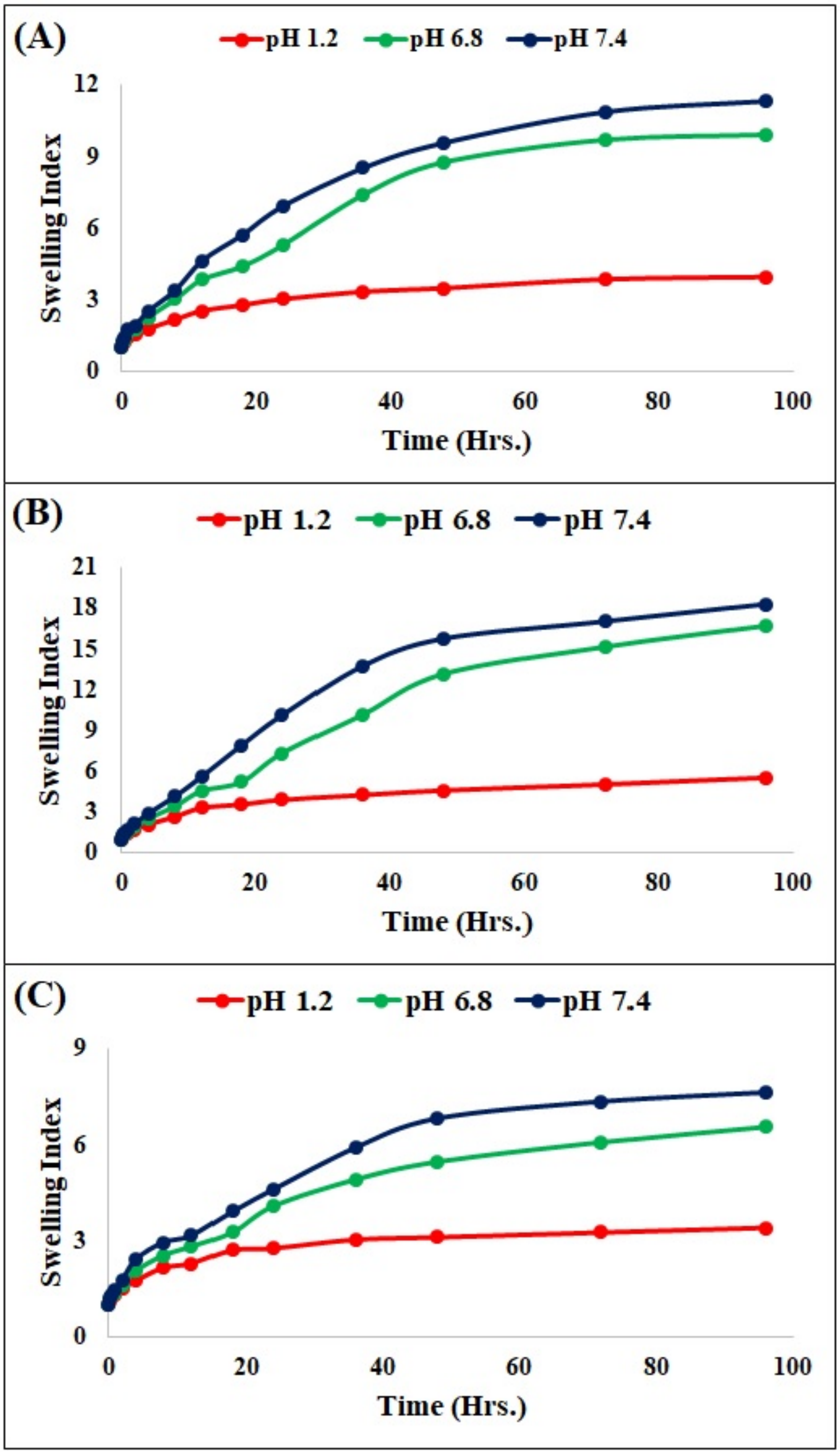

Fig. (S3). Swelling index of formulation MA-3, PU-3 and CR-3.

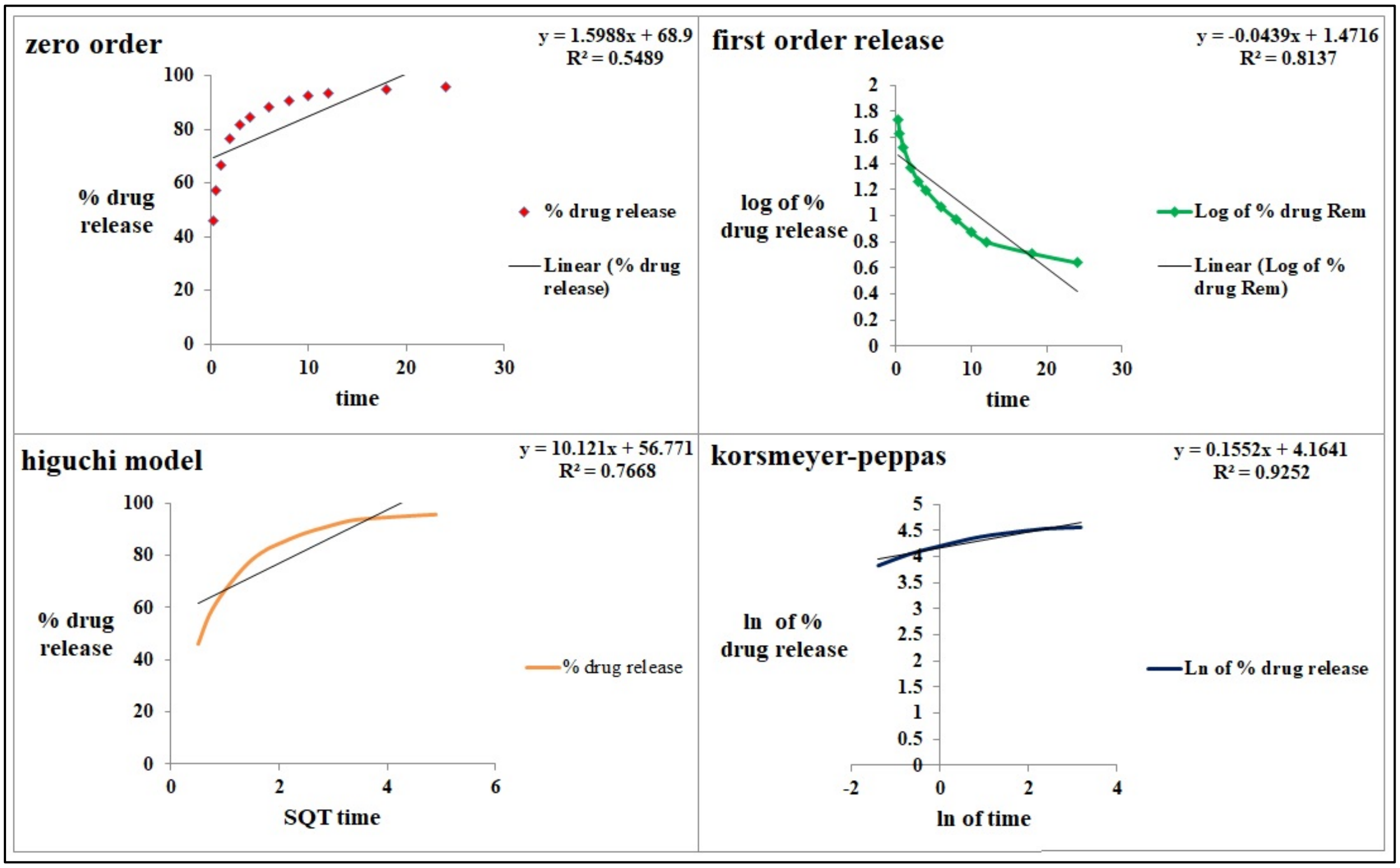

Fig. (S4). Graphical representation of kinetic models.

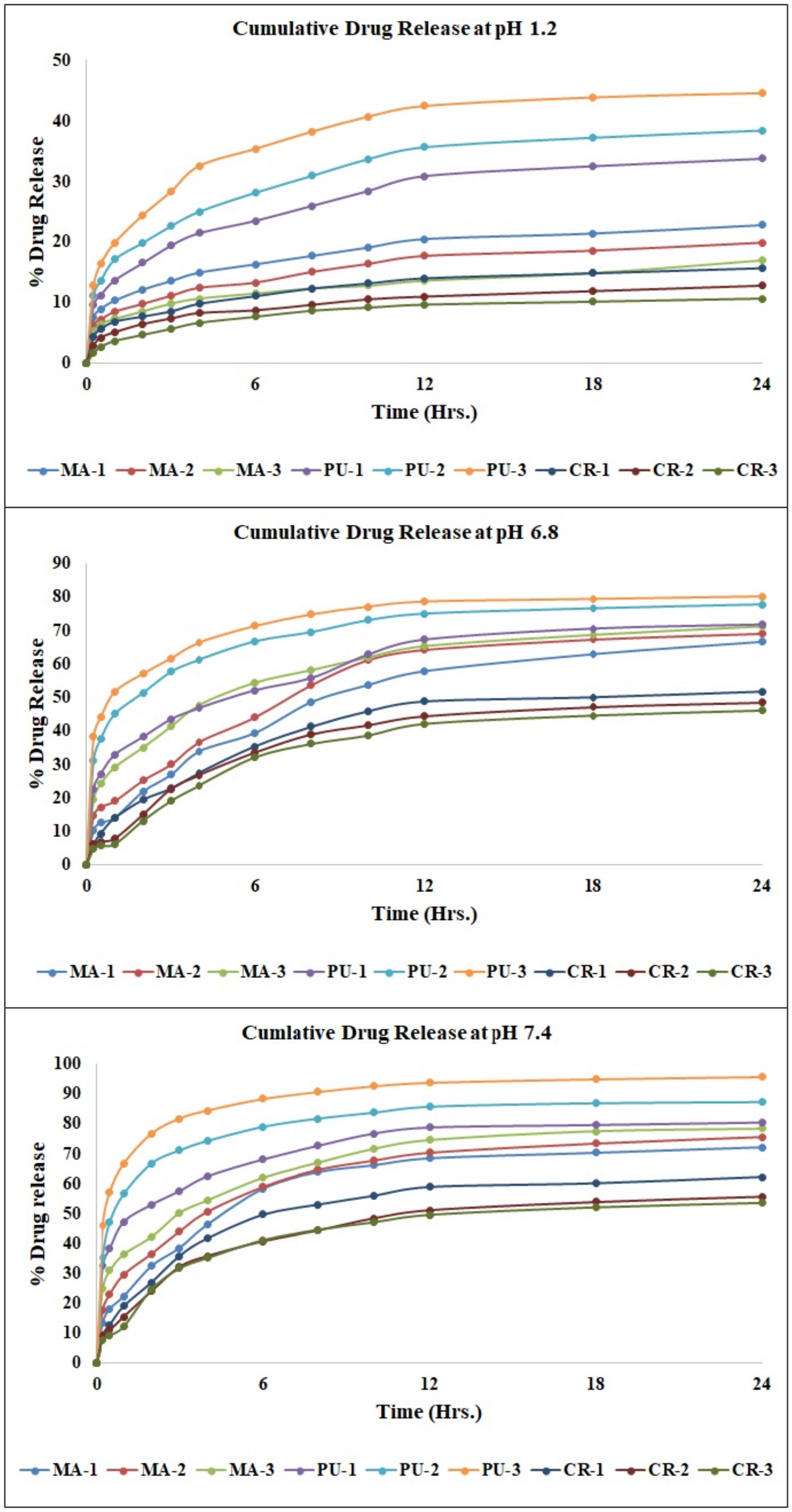

Fig. (S5). Cumulative drug release at pH 1.2, 6.8 and 7.4.

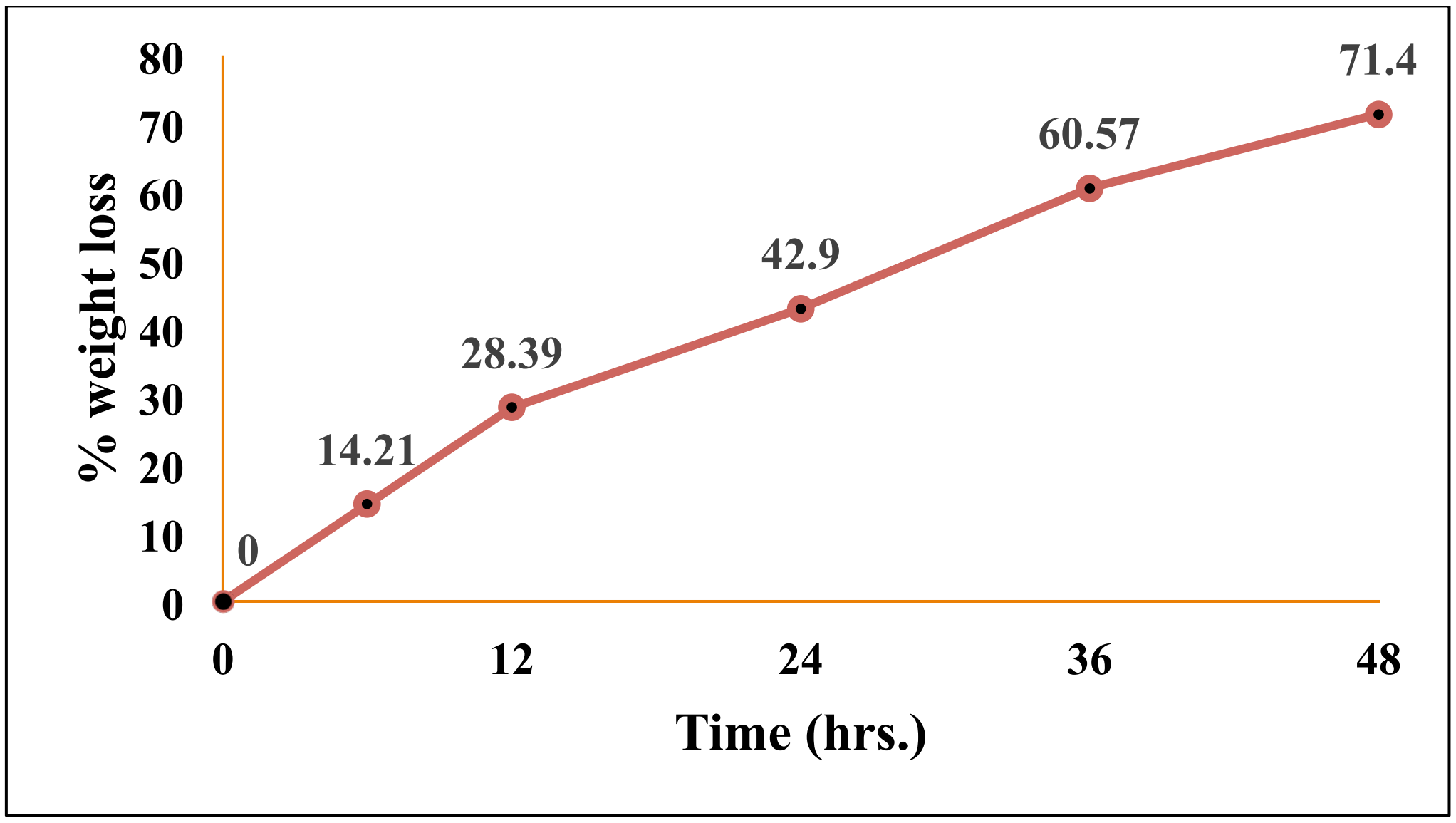

Fig. (S6). Erosion (%) of formulation MA-3.
